# Effect of a Nutraceutical Combination on Oxidative Stress Biomarkers in Healthy Subjects and Patients with Alzheimer’s Disease

**DOI:** 10.3390/nu18050789

**Published:** 2026-02-27

**Authors:** Rafał Jastrząb, Andrzej Małecki, Elżbieta Kmiecik-Małecka, Agnieszka Gorzkowska, Kamil Kubas, Justyna Widłak-Kargul, Damian Wolman, Katarzyna Matkiewicz, Marta Nowacka-Chmielewska, Daniela Liśkiewicz, Konstancja Grabowska, Mateusz Grabowski, Natalia Pondel, Gabriela Początek, Gabriela Kłodowska, Jennifer Mytych

**Affiliations:** 1 Research and Development Center, Olimp Laboratories Sp. z o.o., 39-200 Dębica, Poland; r.jastrzab@olimp-labs.com (R.J.); k.kubas@olimp-labs.com (K.K.); j.widlak@olimp-labs.com (J.W.-K.); d.wolman@olimp-labs.com (D.W.); k.matkiewicz@olimp-labs.com (K.M.); 2 Laboratory of Molecular Biology, Institute of Physiotherapy and Health Sciences, Academy of Physical Education, 40-065 Katowice, Poland; a.malecki@awf.katowice.pl (A.M.); e.kmiecik@wss5.pl (E.K.-M.); m.nowacka@awf.katowice.pl (M.N.-C.); daniela.liskiewicz@gmail.com (D.L.); k.grabowska@awf.katowice.pl (K.G.); m.grabowski@awf.katowice.pl (M.G.); n.pondel@awf.katowice.pl (N.P.); 3 Neurorehabilitation Ward, Silesian Voivodship Hospital No 5, 41-200 Sosnowiec, Poland; 4 Department of Neurology, School of Health Sciences, Medical University of Silesia in Katowice, 40-007 Katowice, Poland; agorzkowska@sum.edu.pl; 5 Department of Neurology, Faculty of Health Sciences in Katowice, Medical University of Silesia, 40-007 Katowice, Poland; d201180@365.sum.edu.pl; 6 Doctoral School of Medical and Health Sciences, Silesian Medical University, 40-007 Katowice, Poland; 7 Neuro-Care Medical Center, 40-749 Katowice, Poland; g.klodowska@neuro-care.pl

**Keywords:** neurodegeneration, aging, Alzheimer’s disease, oxidative stress, phytocomplex

## Abstract

Background/Objectives: Advanced glycation end products (AGEs) and oxidative stress increase with aging and are implicated in Alzheimer’s disease (AD). We developed an anti-glycation blend using LC-MS-based screening and assessed its effects on oxidative and glycation-related biomarkers in humans. Methods: Twelve candidate compounds were screened in a BSA–glucose model using LC-MS peptide mapping to quantify lysine glycation and rank inhibitory activity. The top candidates were combined into a three-compound blend (quercetin, rutin, genistein). In a randomized, double-blind, placebo-controlled 3-month trial, older healthy adults (n = 30) and individuals with AD (n = 30) received anti-AGE blend (n = 15 in older group and n = 15 in AD group) or placebo (n = 15 in older group and n = 15 in AD group). Serum malondialdehyde and urinary Nε-(carboxymethyl)lysine were measured pre–post intervention. Pre/post and between-arm comparisons within each population were performed using REML ANOVA with Tukey post hoc tests. Serum MDA (malondialdehyde) and urinary CML (Nε-(carboxymethyl)lysine) were prespecified biomarker outcomes and are reported here as co-primary biomarker endpoints. No formal a priori sample size calculation was performed; the study size was feasibility-based. Results: LC-MS screening identified genistein, quercetin, and rutin as the most consistent inhibitors of glucose-driven BSA glycation. In older healthy adults, serum MDA decreased after anti-AGE supplementation (*p* < 0.001) and differed from the placebo (*p* < 0.01), while no change was observed within the placebo group (ns). In the AD cohort, MDA did not change significantly from baseline within either arm (ns), but post-intervention MDA was lower in anti-AGE than in the placebo (*p* < 0.05). Urinary CML was unchanged in older healthy adults (ns in both arms), whereas in AD, it decreased after anti-AGE supplementation (*p* < 0.01) and differed from the placebo (*p* < 0.05). Conclusions: A screening-guided anti-glycation blend supplementation was associated with changes in selected biomarkers in humans: MDA decreased across cohorts, while CML decreased selectively in AD. Larger trials with extended biomarker panels and LC–MS/MS confirmation are warranted.

## 1. Introduction

Advanced glycation end products (AGEs) and lipid peroxidation accumulate with aging and are increasingly implicated in age-related disorders, including Alzheimer’s disease (AD) [1]. In both aging and AD, oxidative stress and glyco-oxidative processes may amplify one another, contributing to protein and lipid damage and potentially reducing cellular resilience [2,3].

Among AGEs, Nε-(carboxymethyl)lysine (CML) is a widely used marker of systemic glycation-related burden [4], while malondialdehyde (MDA) is commonly used as an index of lipid peroxidation [5]. From a translational perspective, nutraceutical strategies that can modulate these pathways are attractive because they are generally scalable and well tolerated [6]; however, human evidence remains heterogeneous and often disconnected from a quantitative screening rationale [7].

Previous human intervention studies suggest that nutraceuticals with antioxidant and/or anti-glycation potential can influence oxidative stress and AGE-related biomarkers, but findings are heterogeneous across populations and formulations. Quercetin has been evaluated most often in exercise-challenge cohorts, where short-term supplementation was reported to attenuate lipid peroxidation markers such as malondialdehyde (MDA) during recovery [8]. In metabolic-risk populations, genistein supplementation in a randomized, double-blind, placebo-controlled trial reduced serum MDA and improved antioxidant indices [9]. In contrast, a human supplementation study of rutin showed increased circulating flavonoid exposure without significant change in urinary MDA, underscoring variability related to baseline status and biotransformation [10]. Complementary evidence that glycation-related readouts are responsive comes from controlled low-AGE dietary interventions that quantified changes in plasma and urinary AGEs (including CML) using mass spectrometry, highlighting the influence of exposure and clearance on AGE biomarkers [11]. Together, these data support biological plausibility but also motivate mechanistically guided candidate selection—an unmet gap addressed here.

In this study, we applied a mass spectrometry-based in vitro screening assay using glycated albumin to identify the most active anti-glycation candidates and formulated a three-compound blend (quercetin, rutin, genistein). We then evaluated its effects in a placebo-controlled human study in older healthy adults and individuals with AD, focusing on circulating MDA and urinary CML as complementary readouts of oxidative stress and glycation-related burden.

## 2. Materials and Methods

### 2.1. Anti-Glycation Assay

Anti-glycation activity of selected compounds (trans-ferulic acid, pyridoxine HCl, sodium pyruvate, genistein, quercetin, rutin, epigallocatechin gallate (EGCG), chlorogenic acid, L-arginine, L-citrulline, β-alanine) was evaluated in a bovine serum albumin (BSA) glycation model. BSA (5% *w*/*v*) was incubated for 14 days at 37 °C, with or without glucose (200 mg/dL), in the presence of each candidate compound. After incubation, proteins were digested with trypsin (V5280; Promega, Madison, WI, USA) and analyzed using the nanoLC–MS/MS (nanoAcquity UPLC, Waters, Milford, MA, USA) coupled to an Orbitrap Q Exactive mass spectrometer (Thermo Fisher Scientific, Waltham, MA, USA) operated in data-dependent MS/MS mode (*m*/*z* 300–2000). Raw files were processed in MaxQuant (https://maxquant.org/; 2018–2019) using a UniProt protein database, with variable modifications including lysine glycation (Hex-related), methionine oxidation, and N-terminal acetylation (FDR 1%). Glycation-associated peptide signals were filtered and aligned; results for each peptide were normalized to the no-glycation condition (no-Hex baseline background) and expressed relative to the glucose condition (log ratio), enabling ranking of candidates and selection of the top-performing compounds for formulation.

**Figure 1:**
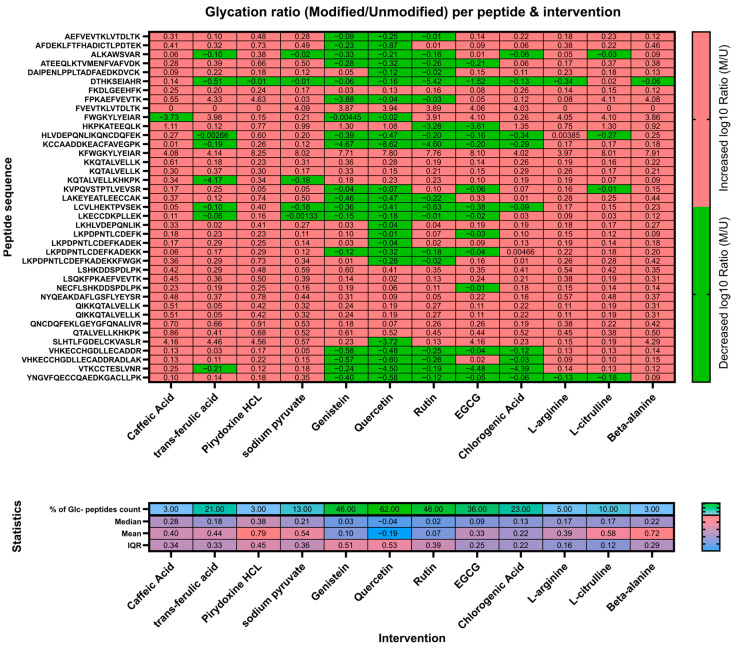
Upper heat-map: screening of 12 candidate compounds for inhibition of BSA glycation assessed by LC–MS peptide mapping. The log ratio change has been calculated between modified (glycated lysin) and unmodified peptide obtained from incubation with particular intervention and glucose solution (200 mg/L) and normalized to the control (log ratio of M/U BSA peptides incubated with just glucose solution). Lower heat-map: in Statistics, the heat-map percentage count ratio between peptides where glycation was suppressed and total modified peptides per compound (% of Glc- peptides count), median, mean and IQR have been provided. Abbreviations: HCL—hydrochloride; EGCG—epigallocatechin gallate.

Candidate compounds were ranked using peptide-level glycation readouts derived from LC–MS peptide mapping. For each quantified lysine-containing peptide, glycation was expressed as a modified/unmodified (M/U) ratio (based on LC–MS signal intensities of the glycated vs. the corresponding unmodified peptide form). For each intervention, peptide-level ratios were log10-transformed and compared to the glucose-only condition; negative values indicate suppression of glucose-driven glycation. For each compound, we summarized: (1) the median peptide-level log10 ratio across all quantified glycation-associated peptides, (2) the interquartile range (IQR), and (3) the number (and proportion) of peptides showing decreased vs. increased glycation relative to glucose-only. Compounds were prioritized based on broad, consistently negative distributions across peptides (i.e., suppression across multiple peptides rather than isolated peptide effects), and the top-performing candidates were selected for formulation. The complete compound ranking output (median, IQR, and peptide counts/proportions per compound) is provided in Figure 1. Peptides not consistently quantified across conditions were excluded from ranking (and treated as missing values without imputation).

### 2.2. UHPLC-PDA Analysis of Quercetin, Rutin and Genistein

Quercetin, rutin and genistein in the study formulation were verified by UHPLC-PDA (Shimadzu Nexera X2, PDA detector; Shimadzu, Kyoto, Japan) on a Kinetex C18 column (150 × 3.0 mm, 2.6 μm; Phenomenex; Torrance, CA, USA) using water/acetonitrile/formic acid mobile phases and a rapid gradient (total run time 7 min; flow 0.8 mL/min; 25 °C; 2 μL injection; detection 260 nm). One-point external calibration was prepared from certified/analytical standards [rutin trihydrate, HWI (Rülzheim, Germany); quercetin dihydrate CRS, EDQM (Strasbourg Cedex, France); genistein, Supelco (Darmstadt, Germany)]. Formulation samples were extracted in water:methanol, clarified by centrifugation, diluted and injected for quantification/verification.

### 2.3. Clinical Study Design, Participants, and Intervention

A randomized, double-blind, placebo-controlled trial with pre–post assessments was conducted in two populations, both predominantly female: older healthy adults and patients with early-stage Alzheimer’s disease (AD). Thirty healthy participants and thirty AD participants were enrolled and randomized 1:1 within each population to receive the anti-AGE formulation or placebo for 3 months. Healthy eligibility: 55–85 years, no diagnosis of AD or Parkinson’s disease, and MMSE > 24. AD eligibility: early-stage AD per NINCDS-ADRDA/NIA-AA criteria, MMSE > 24. Key exclusions for both populations included diabetes, active substance abuse, unstable ischemic heart disease, uncontrolled hypertension, history of severe allergic reactions, major conditions affecting safety/participation, and use of psychotropic drugs. To reduce variability in oxidative- and glycation-related biomarkers, participants were instructed to maintain a stable habitual diet for at least 3 months prior to enrollment and throughout the intervention period. During the study, participants were not allowed to initiate any new dietary supplements. Participants were also instructed not to introduce major dietary changes during the intervention. Concomitant medications were recorded at baseline and participants were encouraged to keep chronic medication regimens stable unless medically indicated. The study was approved by the relevant Bioethics Committee (Resolution No. 6/2022; 23 June 2022). Detailed characterization of the healthy and AD study arm is presented in Table 1.

Participants were randomized with computer-generated permuted blocks (block size 4). Anti-AGE capsules contained quercetin (1000 mg/day as active ingredient), rutin (50 mg/day), and genistein (50 mg/day) plus excipients; placebo capsules were identical in appearance and contained excipients only.

Dose selection and bioavailability considerations: the daily dose of quercetin (1000 mg/day) was selected to support sufficient systemic exposure, given its generally low oral bioavailability and extensive first-pass metabolism. In human supplementation studies, quercetin doses in the range of ~500–1000 mg/day are commonly used and are typically well tolerated. Rutin (50 mg/day) and genistein (50 mg/day) were administered at doses consistent with commonly used nutraceutical ranges and were selected based on their robust anti-glycation performance in the LC–MS screening step. Thus, the formulation ratio reflects an exposure- and tolerability-oriented strategy rather than an assumption of equal potency by mass. Dose selection also reflected EU/Poland dietary-supplement safety guidance (including conservative dosing for isoflavones), supporting the genistein dose (50 mg/day), whereas quercetin was dosed higher to compensate for limited bioavailability. In addition, rutin (a quercetin glycoside) may contribute via distinct absorption kinetics and enzymatic/microbiota-mediated conversion to quercetin-derived metabolites.

Products were manufactured and quality-verified by Olimp Laboratories (Dębica, Poland). Participants and study staff remained blinded until study completion.

### 2.4. Biochemical Outcomes

Malondialdehyde (MDA) was quantified in serum using a competitive colorimetric ELISA (ab287797, Abcam, Cambridge, UK). Briefly, standards and appropriately diluted serum samples were added to precoated microplate wells together with kit reagents according to the manufacturer’s protocol, incubated to allow competitive binding, and subsequently washed to remove unbound material. The colorimetric signal was developed using the kit substrate solution and absorbance was measured at 450 nm using a microplate reader. Concentrations were calculated from the standard curve and are reported as immunoreactive MDA equivalents within the manufacturer-stated assay performance characteristics (Table S1).

Urinary Nε-(carboxymethyl)lysine (CML) was quantified using a competitive ELISA kit (UNEB0082, Assay Genie, Dublin, Ireland). Briefly, standards and appropriately diluted urine samples were added to CML-precoated microplate wells together with kit detection reagents according to the manufacturer’s protocol, incubated at 37 °C, and washed between steps. The colorimetric signal was developed using TMB substrate and absorbance was measured at 450 nm using a microplate reader. Concentrations were calculated from the plate-specific standard curve (4-parameter logistic fit) and corrected for dilution factors. The manufacturer-stated assay range was 0.78–50 ng/mL; samples exceeding the upper limit were re-assayed after further dilution to bring interpolated values within the validated range and final concentrations were obtained by multiplying by the corresponding dilution factor. The kit is a competitive immunoassay using an antibody described by the manufacturer as specific to CML; therefore, results are reported as immunoreactive CML equivalents within the assay’s stated performance characteristics (Table S1).

### 2.5. Statistical Analyses

MDA and CML were analyzed using linear mixed-effects models fitted by REML, including fixed effects for group (placebo vs. anti-AGE), time (pre vs. post), and their interaction (group × time), with subject-specific random intercepts. Baseline values were flexibly adjusted using a spline term that interacted with the group. The prespecified estimand was the difference-in-differences (group × time), obtained from estimated marginal means with Kenward–Roger degrees of freedom. Sensitivity analyses used change scores (post–pre) compared between arms (Welch’s *t*-test). Model diagnostics were assessed using DHARMa. Analyses were conducted in R version 4.5.2 (lme4, lmerTest, emmeans, splines, DHARMa); two-sided α = 0.05. All boxplot charts were created using ggplot2 R package.

## 3. Results

### 3.1. Proteomic Profiling of BSA Glycation Products

Because AGE formation largely reflects lysine modifications, we performed an in vitro LC-MS peptide-mapping screen in a BSA–glucose model to rank 12 candidate compounds by their ability to suppress glucose-driven glycation and to localize responsive glycation sites. This screening identified clear compound-dependent differences in glycation patterns (Figure 1) and was used to select candidates for subsequent formulation and clinical testing.

Across lysine-containing peptides, glucose alone induced broad increases in glycation. Among tested compounds, genistein, quercetin, and rutin showed the most consistent suppression of glycation signals across peptide sequences, reflected by predominantly negative log ratios (green) and the lowest median glycation values (−0.094, −0.009, and −0.001, respectively). These conditions also yielded the fewest peptides with increased glycation (9–17 peptides), whereas several other candidates showed weaker or inconsistent effects, with higher median scores (0.005–0.052) and more peptides exhibiting increased glycation (25–33 peptides). Collectively, the LC-MS mapping supported genistein, quercetin, and rutin as the most robust anti-glycation candidates in this model (Figure 1).

In addition to visual inspection of peptide-level patterns (Figure 1), we quantified compound performance using distribution-based summary metrics (median and IQR of peptide-level log ratios) and peptide counts indicating directionality (number of peptides with reduced vs. increased glycation relative to glucose-only). This approach allowed us to prioritize compounds showing broad, consistent suppression of glycation signals across multiple lysine-containing peptides rather than isolated peptide effects.

### 3.2. UHPLC-PDA Analysis of Tested Blend

**Figure 2:**
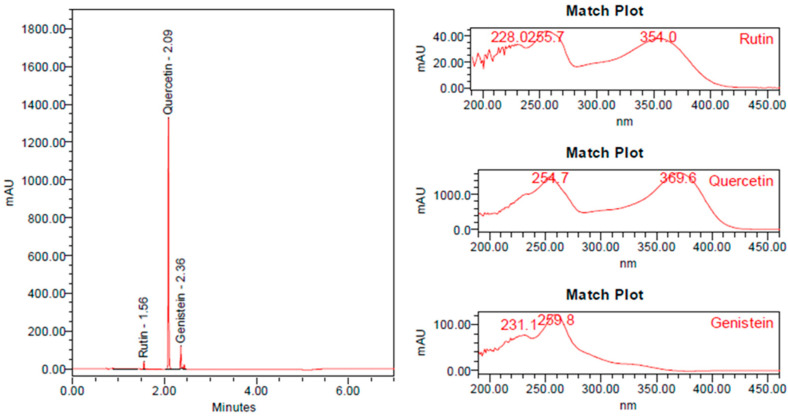
PDA chromatogram and spectra for rutin, quercetin, and genistein in test solution.

The chromatogram and PDA spectrum for the blend test solution showed peaks corresponding to three analytes: rutin at a retention time of 1.56 min, quercetin at a retention time of 2.09 min and genistein at a retention time of 2.36 min. All those obtained from the corresponding standard solutions. In addition, the PDA spectra of the test solution were compared and showed maxima at wavelengths comparable to those of the standard solutions. The created formulation was tested for raw material identity (Figure 2).

### 3.3. Malondialdehyde (MDA) Levels in Healthy Subjects and Alzheimer’s Patients

**Figure 3:**
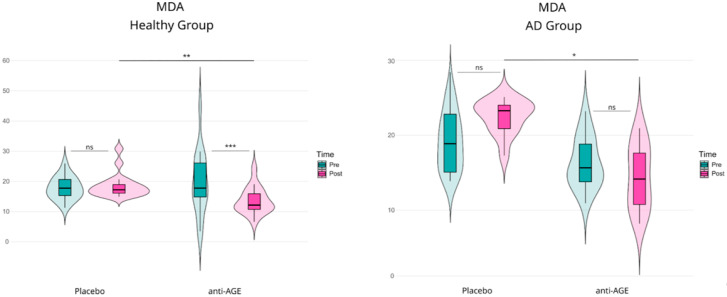
Levels of MDA in serum of patients after 3 months’ supplementation with anti-glycation blend (anti-AGE). Pre/Post comparisons of MDA between placebo and anti-AGE group in each study population (healthy, AD) have been made using REML ANOVA with Tukey post hoc comparison; outliers have been filtered using the IQR method (n-Placebo-Healthy: 15, n-anti-AGE-Healthy: 15; n-Placebo-AD: 12, n-anti-AGE-AD: 14). Significance code: ns—non significant, *—*p* < 0.05, **—*p* < 0.01, ***—*p* < 0.001.

Oxidative stress contributes to both aging and neurodegeneration, and malondialdehyde (MDA) is commonly used as a marker of lipid peroxidation. To assess whether the anti-glycation blend exerts antioxidant effects in vivo, serum MDA was measured pre- and post-intervention in older healthy adults and individuals with AD (Figure 3). Because oxidative and glycation-related pathways are closely linked, MDA assessment was complemented by urinary CML analysis (see below).

In 17. 79 ± 3.76 ng/mL vs. 18.62 ± 4.07 ng/mL; Hedges’ g: 0.2; ns). In contrast, participants receiving anti-AGE showed a statistically significant reduction in MDA from pre- to post-intervention (Pre vs. Post: 19.66 ± 10.48 ng/mL vs. 13.58 ± 4.33 ng/mL; Hedges’ g: −0.74; *p* < 0.001; ***), indicating a reduction in this lipid peroxidation marker following supplementation. Between-arm post hoc comparisons additionally supported a intervention effect in the healthy group, with anti-AGE differing significantly from the placebo (Placebo vs. Intervention: 18.62 ± 4.07 ng/mL vs. 13.58 ± 4.33 ng/mL; Hedges’ g: −1.14; *p* < 0.01; **). In the AD population, MDA levels increased over the study period in the placebo arm (Pre vs. Post: 19.74 ± 4.82 ng/mL vs. 22.49 ± 2.26 ng/mL; Hedges’ g: 0.7; ns). In contrast, within the anti-AGE arm, the pre/post difference indicated a reduction in MDA levels (Pre vs. Post: 16.33 ± 3.42 ng/mL vs. 14,47 ± 4.28 ng/mL; Hedges’ g: 0.4; ns). However, the mentioned changes did not reach the threshold for statistical significance (ns). Interestingly, the between-arm post hoc comparison indicated lower MDA in the anti-AGE arm compared with the placebo (Placebo vs. Intervention: 22.49 ± 2.26 ng/mL vs. 14.47 ± 4.28 ng/mL; Hedges’ g: −2.3; *p* < 0.05; *), consistent with a placebo-adjusted treatment effect despite the lack of a statistically significant within-arm pre/post change.

### 3.4. Carboxymethyllysine (CML) Levels in Healthy Subjects and Alzheimer’s Patients

**Figure 4:**
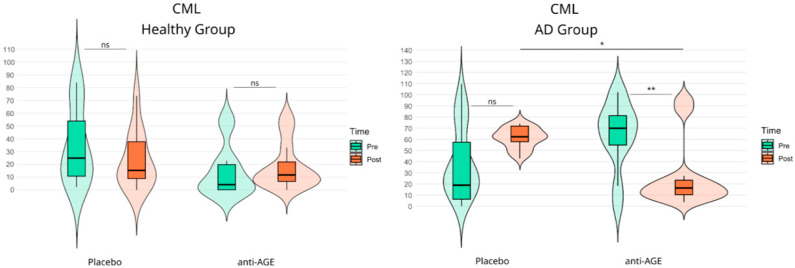
Levels of CML in urine of patients after 3 months’ supplementation with anti-glycation blend (ant-AGE). Pre/post comparisons of CML between placebo and anti-AGE group in each study population (healthy, AD) have been made using REML ANOVA with Tukey post hoc comparison; outliers have been filtered using the IQR method (CML: n-Placebo-Healthy: 11, n-anti-AGE-Healthy: 11; CML: n-Placebo-AD: 8, n-anti-AGE-AD: 8). Significance code: ns—non significant, *—*p* < 0.05, **—*p* < 0.01.

Advanced glycation end products also accumulate with aging and have been implicated in neurodegeneration. Nε-(carboxymethyl)lysine (CML) is a widely used AGE marker of systemic glycation-related burden. Therefore, to complement the oxidative stress readout (MDA), we measured urinary CML pre–post intervention to capture potential treatment effects on glycation-related processes (Figure 4).

In the healthy population, urinary CML did not change significantly from pre- to post-intervention in either the placebo arm (Pre vs. Post: 33.69 ± 29.57 ng/mL vs. 23.09 ± 22.01 ng/mL; Hedges’ g: −0.39; ns) or the anti-AGE arm (Pre vs. Post: 14.56 ± 19.91 ng/mL vs. 18.46 ± 18.18 ng/mL; Hedges’ g: 0.19; ns), indicating no detectable time-dependent effect of supplementation on this AGE marker in older healthy adults. In the AD population, urinary CML showed no significant pre/post increase in the placebo arm (Pre vs. Post: 37.92 ± 47.74 ng/mL vs. 60.49 ± 10.87 ng/mL; Hedges’ g: 0.63; ns). In contrast, participants receiving anti-AGE exhibited a statistically significant reduction in urinary CML from pre to post (Pre vs. Post: 62.96 ± 31.46 ng/mL vs. 20.80 ± 22.73 ng/mL; Hedges’ g: −1.48; *p* < 0.01; **), consistent with a marked decrease in glycation-related burden following supplementation. In addition, the between-arm post hoc comparison indicated a difference between anti-AGE and placebo within the AD cohort (Placebo vs. Intervention: 60.49 ± 10.87 ng/mL vs. 20.80 ± 22.73 ng/mL; Hedges’ g: −2.20; *p* < 0.05; *; for all CIs, check Supplementary Table S2), supporting a placebo-adjusted treatment effect on urinary CML.

## 4. Discussion

In this placebo-controlled human study conducted in older healthy adults and individuals with Alzheimer’s disease (AD), a rationally selected three-compound blend (quercetin, rutin, genistein) was associated with consistent changes in selected oxidative and glycation-related readouts [1,12]. The most consistent signal was observed for serum MDA [13], which decreased following supplementation in the healthy cohort and differed from the placebo in both populations. Notably, the AGE-related biomarker urinary CML [14] was unchanged in older healthy adults but was significantly reduced in AD, both over time and relative to the placebo. Together, these findings support the concept that targeting glyco-oxidative processes with a screening-guided nutraceutical formulation may be associated with changes in selected biomarker readouts in humans, with the magnitude of response depending on baseline metabolic burden [12].

Bioavailability is an important consideration for polyphenols and isoflavones. Quercetin is absorbed to a limited extent and circulates largely as phase II conjugates, whereas rutin (a quercetin glycoside) exhibits different absorption kinetics and is partly converted by intestinal enzymes and gut microbiota, potentially contributing additional quercetin-derived metabolites. Genistein is also extensively metabolized, and its circulating forms may differ from the parent compound. In the present study, we did not quantify circulating levels of the three compounds or their metabolites; therefore, future trials should incorporate pharmacokinetic measurements (preferably LC–MS/MS) to link exposure to biomarker responses, better interpret between-group differences, and refine dose ratios.

A key observation is the population-specific pattern for CML. The absence of change in older healthy adults may reflect a floor effect or limited short-term dynamics of CML when systemic glycation burden is comparatively lower and more stable. In contrast, AD participants may present with a higher baseline “glyco-oxidative load” [15] and/or greater ongoing carbonyl stress [16], providing more room for improvement and making supplementation effects detectable. This interpretation is consistent with the broader view that aging-related glycation and oxidation can be present in both groups, while AD may represent a state of amplified dysregulation. Importantly, the divergence between MDA (responsive in both groups) and CML (responsive predominantly in AD) also suggests that the intervention may more readily affect lipid peroxidation pathways across aging phenotypes, whereas a measurable impact on AGE burden may require a higher baseline glycation-related disturbance [3].

While the population-specific CML pattern is consistent with differences in baseline glyco-oxidative burden, alternative explanations should also be considered. Even with instructions to maintain stable dietary habits and not to initiate new supplements, residual differences in dietary composition (including dietary AGE exposure) and day-to-day intake can influence oxidative and glycation-related readouts. Concomitant medication use—particularly common in older and AD cohorts—may affect redox balance, lipid peroxidation, and glycation pathways and could contribute to between-individual variability. For urinary CML, hydration status and renal handling (including variations in urine concentration and renal function) can materially influence measured concentrations; thus, future studies should incorporate normalization strategies (e.g., creatinine), capture renal function indices, and consider repeated sampling to better quantify intra-individual biological variability.

Mechanistically, the observed biomarker shifts are plausible given the known biochemical properties of the selected compounds. Quercetin and rutin [17] (as a quercetin glycoside) and genistein [18] have been linked to antioxidant activity, modulation of redox-sensitive signaling, and potential interference with glycation chemistry via carbonyl trapping, metal chelation, and suppression of oxidative steps that propagate AGE formation [19,20]. The blend was not chosen empirically; rather, it was derived from a mass spectrometry-based in vitro screening in a glycated-albumin model. This “rational selection” step may be particularly relevant in a field where heterogeneous clinical results are often attributed to variable compound choice, dosing, and bioavailability. Additionally, rutin and genistein undergo extensive metabolism, and their circulating/urinary metabolites may contribute to systemic effects, which could partially explain why changes were observed in both serum (MDA) and urine (CML), albeit in a population-dependent manner.

The BSA–glucose incubation assay models an early-stage protein glycation context under controlled conditions and is useful for comparative ranking of candidate inhibitors at the protein/peptide level. However, it does not capture key determinants of AGE regulation in humans, including digestion and absorption, extensive phase II metabolism and microbial conversion of polyphenols, tissue distribution, endogenous carbonyl-stress sources (e.g., methylglyoxal), and renal clearance of AGE adducts. Therefore, the LC–MS screen was used as a selection tool to prioritize compounds with consistent anti-glycation signatures, rather than as a direct quantitative predictor of clinical biomarker changes. Importantly, the screening assay was not intended to predict the magnitude of biomarker changes in vivo, but to reduce arbitrariness in compound selection by enriching for candidates with consistent anti-glycation signatures at the peptide level. The observed human readouts (MDA and urinary CML) are downstream, exposure- and clearance-dependent measures, and their responsiveness is shaped by absorption, metabolism, microbiota-mediated conversion, and renal handling—processes not captured by the in vitro model. Accordingly, future work should integrate LC–MS/MS exposure measurements and broader AGE/carbonyl-stress panels to strengthen mechanistic bridging.

Several limitations should be considered. First, as a short communication with a limited biomarker panel and modest sample size, the present results should be interpreted as biological signals rather than definitive clinical efficacy. Second, both endpoints were quantified by immunoassays; while suitable for within-study comparisons, ELISA-based readouts can be sensitive to pre-analytical handling and may not match the molecular specificity of chromatographic methods [21]. Third, urinary biomarkers are inherently influenced by urine concentration and renal handling; therefore, normalization (e.g., to creatinine) and accounting for renal function are important considerations for future work [22]. Finally, although participants were instructed to keep their diet stable (including no major dietary changes for ≥3 months before enrollment and during the intervention) and not to initiate new supplements, dietary AGE exposure was not quantified; together with metabolic comorbidities and medication use—particularly relevant in older and AD cohorts—these factors could contribute to inter-individual variability and should be systematically captured and modeled in larger studies.

Despite these constraints, the pattern of results—MDA reduction across cohorts and CML reduction selectively in AD—is internally coherent and supports a testable hypothesis: a screening-guided anti-glycation formulation may exert broad antioxidant effects in older adults, while potentially biologically meaningful reductions in AGE burden may be most apparent in populations with higher baseline glycation-related stress [1]. Future studies should extend the intervention period, incorporate additional AGE and carbonyl-stress markers (e.g., CEL, MG-H1, methylglyoxal-related adducts), use LC-MS/MS confirmation for key analytes, and evaluate functional outcomes relevant to aging and AD. Such work would clarify whether the observed biomarker shifts reflect durable changes in glyco-oxidative biology and whether they are associated with improvements in outcomes relevant to aging and AD.

## 5. Patents

We registered P.452927: “Pharmaceutical composition for improving the general condition of patients with a neurodegenerative disease”.

## Figures and Tables

**Table 1:** Baseline characteristics of individuals.

Group	Healthy	Alzheimer’s Disease
Sample size (n)	30	30
Sex [% of woman]	74.5	77.4
Age	75.7 ± 8.7	83.1 ± 8.6
ALT [U/L]	18.47 ± 11.11	12.98 ± 5.16
AST [U/L]	21.68 ± 11.14	20.36 ± 6.17
Bilirubin [mg/dL]	0.73 ± 0.33	0.68 ± 0.22
CRP [mg/L]	3.54 ± 5.50	8.79 ± 15.64
Glucose [mg/dL]	101.86 ± 24.44	101.82 ± 15.64
HbA1c [%]	5.86 ± 0.87	5.87 ± 0.86
Insulin [µIU/mL]	8.00 ± 4.63	11.47 ± 10.35
Creatinine [mg/dL]	1.03 ± 0.67	0.90 ± 0.26

Abbreviations: ALT—alanine aminotransferase; AST—aspartate aminotransferase; CRP—C-reactive protein; HBA1c—glycated hemoglobin.

## Data Availability

The datasets generated and analyzed during the current study contain sensitive medical information. In accordance with GDPR regulations, patient confidentiality standards, and the requirements of the local ethics committee, these data cannot be made publicly available. However, all relevant findings are transparently presented in the manuscript enabling independent assessment without requiring access to individual-level medical records. For scientifically justified and ethically sound inquiries, access to anonymized datasets may be granted upon reasonable request to the corresponding author [JM], provided that appropriate safeguards for data protection and confidentiality are in place.

## Supplementary Materials

The following supporting information can be downloaded at https://www.mdpi.com/article/10.3390/nu18050789/s1, Table S1: ELISA assay details and manufacturer-reported performance characteristics for MDA and CML measurements, Table S2: Confidence intervals for all groups and timepoints.

## Abbreviations

The following abbreviations are used in this manuscript:

AGEs	Advanced glycation end products
AD	Alzheimer’s disease
CML	Nε-(carboxymethyl)lysine
MDA	Malondialdehyde
EGCG	Epigallocatechin gallate
BSA	Bovine serum albumin
ALT	Alanine aminotransferase
AST	Aspartate aminotransferase
CRP	C-reactive protein
HBA1c	Glycated hemoglobin
HCL	Hydrochloride
