# Supplementary material for: Effect of a Nutraceutical Combination on Oxidative Stress Biomarkers in Healthy Subjects and Patients with Alzheimer’s Disease
Source: Nutrients. 2026 Feb 27;18(5):789. doi: 10.3390/nu18050789 (PMC12986708; doi:10.3390/nu18050789)
Supplement: Supplementary file 1 [file nutrients-18-00789-s001.zip › Table S1.pdf]

Table S1. ELISA assay details and manufacturer-reported performance characteristics for MDA and CML measurements.

Biomarker	Kit (manufacturer; catalog no.)	Reactivity	Assay principle / detection	Sample types	Matrix used in study	Range	Sensitivity / LOD	Precision (intra-assay)	Precision (inter-assay)
Malondialdehyde (MDA)	MDA ELISA Kit; (Abcam, ab287797)	Human	Competitive ELISA; readout 450 nm	Plasma, Cell culture supernatant, Serum	Serum	7.813 - 500 ng/mL	<4.688 ng/mL	CV <8%	CV <10%
Nε-(carboxymethyl)lysine (CML)	N(6)-Carboxymethyllysine (CML) ELISA Kit; (Assay Genie UNEB0082)	General	Competitive ELISA; readout 450 nm	Serum, plasma, saliva, urine, cell culture supernatant, tissue samples and other related supernatants	Urine	0.78-50 ng/mL	Lot-specific	Lot-specific	Lot-specific
