# Supplementary material for: Effect of a Nutraceutical Combination on Oxidative Stress Biomarkers in Healthy Subjects and Patients with Alzheimer’s Disease
Source: Nutrients. 2026 Feb 27;18(5):789. doi: 10.3390/nu18050789 (PMC12986708; doi:10.3390/nu18050789)
Supplement: Supplementary file 1 [file nutrients-18-00789-s001.zip › Table S2.pdf]

Table S2. Confidence intervals for all groups and timepoints.

Group	Health Status	Parameter	Time	CI low	CI high
Placebo	AD	CML	Pre	-12,17	88,03
			Post	50,44	70,55
anti-AGE	AD	CML	Pre	41,83	84,1
			Post	5,52	36,07
Placebo	Healthy	CML	Pre	14,9	52,48
			Post	9,1	37,07
anti-AGE	Healthy	CML	Pre	1,91	27,21
			Post	6,91	30,01
Placebo	AD	MDA	Pre	16,68	22,81
			Post	21,05	23,93
anti-AGE	AD	MDA	Pre	14,35	18,3
			Post	11,99	16,94
Placebo	Healthy	MDA	Pre	15,91	19,65
			Post	16,6	20,64
anti-AGE	Healthy	MDA	Pre	14,08	25,25
			Post	11,27	15,89
